# Correspondence

**Published:** 1890-09

**Authors:** T. J. Cook

**Affiliations:** Roby, Texas


ROBY, TEXAS, July 31, 1890.

*Editors Southern Medical Record:*

I send you a report of a case, which I hope will be of some interest to your readers.

Mrs. B., age 35, widow, small built, mother of three children, the eldest is fourteen and the youngest is ten years of age. About six years ago she had what the doctors called billious colic; about a year afterwards she had another spell. She then began to have them more frequently; last fall the spells began coming every two weeks. She moved to this country last winter, since which time I have been treating her, and she has had spells almost regularly every two weeks since.

During the paroxysm I used the usual remedies, such as morphine, hypodermically, inhalations of chloroform, etc. I put her under treatment known as Durand method, with but little or no benefit.

Will some one suggest something through this journal that may be of service to my patient. T. J. COOK, M. D.

